# Social multiplier effects: academics’ and practitioners’ perspective on the benefits of a tuberculosis operational research capacity-building program in Indonesia

**DOI:** 10.1080/16549716.2017.1381442

**Published:** 2017-10-17

**Authors:** Ari Probandari, Yodi Mahendradhata, Bagoes Widjanarko, Bachti Alisjahbana, On behalf of Tuberculosis Operational Research Group (TORG)

**Affiliations:** ^a^ Department of Public Health, Faculty of Medicine, Universitas Sebelas Maret, Surakarta, Indonesia; ^b^ Center for Health Policy and Management, Faculty of Medicine, Universitas Gadjah Mada, Yogyakarta, Indonesia; ^c^ Department of Health Policy and Management, Faculty of Medicine, Universitas Gadjah Mada, Yogyakarta, Indonesia; ^d^ Department of Health Promotion, Faculty of Public Health, Diponegoro University, Semarang, Indonesia; ^e^ Department of Internal Medicine, Faculty of Medicine, Padjajaran University, Bandung, Indonesia

**Keywords:** Qualitative research, content analysis, public health

## Abstract

**Background**: The Tuberculosis Operational Research Group (TORG) implemented a capacity-building model involving academics and practitioners (i.e. clinicians or program staff) in an operational research (OR) team in Indonesia.

**Objective**: This study explored academics’ and practitioners’ perspectives regarding the benefits of participating in a tuberculosis (TB) OR capacity-building program in Indonesia.

**Methods**: We conducted a qualitative study involving in-depth interviews with 36 academics and 23 practitioners undertaking the TORG capacity-building program. We asked open-ended questions about their experience of the program. Data were analyzed via content analysis.

**Results**: The findings demonstrated the social multiplier effects of the OR capacity-building program. Both academics and practitioners reported perceived improvements in research knowledge, skills, and experience, and described additional individual- and institutional-level benefits. The individual-level benefits level included improvements in understanding of the TB program, motivation for research and self-satisfaction, the development/enhancement of individual networking, receipt of recognition, and new opportunities. The additional benefits reported at an institutional level included improvement in research curricula, in-house training, and program management and the development/enhancement of institutional partnerships.

**Conclusions**: The program improved not only individuals’ capacity for conducting OR but also the quality of the TB program management and public health education. OR should be included in research methodology curricula for postgraduate public health/disease control programs. The capacity-building model, in which academics and program staff collaborated within an OR team, should be promoted.

## Background

Operational research (OR) has recently received increased attention because of its contribution to efforts to control tuberculosis (TB) epidemic [1]. OR is expected to provide evidence to support the improvement of the TB control program. In response to the call to strengthen OR regarding TB, there are ongoing international and national initiatives in place to increase OR capacity in countries with high TB burden [2–4]. Indonesia initiated OR capacity building in 2004, via the establishment of the Tuberculosis Operational Research Group (TORG) [5].

There are several OR capacity-building programs, including the Union/Médecins Sans Frontières Structured Operational Research and Training Initiative program [6] and the Technology, Research, Education and Technical Assistance for Tuberculosis project [7], which train mainly practitioners [6]. In addition, some models, such as the Operational Research Assistance Project [7] and the TORG program [5], deliberately combine participants from academia and health services.

Naidoo et al. [4] suggested that evaluation of the benefits of OR capacity building should be comprehensive and include multiple outcomes. Numbers of publications and use of research in policy and program improvements have been highlighted as indicators in the evaluation of OR capacity-building programs [8,9]. As experience plays a major role in knowledge development [10], in-depth assessment of participants’ experience of the benefits of TB-related OR capacity-building programs is a critical evaluation domain. Nevertheless, there is a lack of research examining participants’ in-depth learning evaluations of such programs of OR capacity-building. Therefore, this study aimed to explore academics’ and practitioners’ perspectives regarding the benefits of participating in an OR capacity-building program in Indonesia.

## Methods

### Context

Since 2004, the TORG program has been conducted as an OR capacity-building program for provincial OR teams. The provincial OR team consists of two academics and two–three clinicians or disease control program officers (practitioners). The OR capacity-building program was conducted as a series of workshops over 1.5 years and included the following: a proposal development workshop, project implementation mentoring, a data management and analysis workshop, and a report-writing workshop. The workshops applied experiential learning methods [10] (i.e. abstract conceptualization through lectures, active experimentation through exercises, concrete experience through field data collection, and reflective observation through supervision and mentoring). Details of the program were described in a previous publication [5].

### Study design

This study involved a qualitative research approach. The qualitative research approach was used in the nature of the exploration of various personal experiences [11]. The qualitative study design is relevant to the nature of this study objective that explores subjective experiences of the academics’ and practitioners on the benefit from participating the TORG capacity-building program. This study was conducted as part of the Impact of Operational Research on TB Program Policy and Practice in Indonesia program in 2014–2015. Previous publication about the project can be found elsewhere [9].

### Researchers’ characteristics and reflexivity

Research conducted by the authors during the preceding 10 years had focused on TB. Three of the authors, BW, YM, and BA, had been involved in the TORG since 2004, and AP joined the TORG seven years later. All authors are experienced in conducting qualitative research.

### Units of study and sampling strategy

The units of study were the academics and practitioners (n = 145) who participated in the TORG workshops. We received a list of academics and practitioners from 33 provincial OR groups that participated in TORG workshops from TB Sub-directorate, Ministry of Health Republic of Indonesia. We conducted a purposive sampling to recruit the principal investigator and another co-investigator from each team, who were undertaking the series of TORG capacity-building workshops completely. The rationale of this sampling method was selecting the informants with rich information about their experiences from participating TORG capacity building. Using the list we contacted the potential study participants. Following the qualitative study method, we determined the number of study participants when data was saturated [12].

### Data collection

Data were collected via in-depth interviews. To minimize the bias to the position of the authors as TORG members, a trained research assistant conducted the interviews under the supervision of AP. We developed an interview guide, which consisted of the following questions:

- How would you describe the experience you have gained from your participation in the TORG capacity-building program?
- How did the experience influence your career?
- How did the experience influence your research interests?
- How did the interactions with OR peers influence communication between your relative institutions?

### Data analysis

All interviews, which included the provision of informed consent, were audio recorded. The research assistant wrote verbatim transcripts within 48 hours of each interview. AP checked the accuracy of the verbatim transcripts. AP and BW conducted individual coding of the data. The results of the individual coding were discussed in the data analysis and interpretation with all researchers.

An inductive qualitative content analysis [13,14] was performed using Open Code 4.02 software [15], to explore manifest and latent meanings of the transcript text. Meaning units consisted of portions of text that contained meaning, and they were labeled using codes. The codes consisted of manifest meanings (obvious meaning in any words or phrases in the meaning units) and latent meanings (implicit meaning in the meaning units). Codes with similar meaning units were classified into categories. The link between categories was defined as a theme.

### Trustworthiness

To increase the trustworthiness of the qualitative study [11], we performed triangulation using the information obtained from participants. For instance, we compared the experience from an academic to other academics or from a practitioner with other practitioners. We also conducted a peer-debriefing technique. The coding process and interpretation was discussed during the workshop of the research team as well as being presented with other TORG members.

### Research ethics

We sent emails to participants, to inform them about the study and assure them of their autonomy, prior to initiating the interviews. All of the participants provided verbal consent, which was audio recorded. We maintained participants’ anonymity throughout the interview transcription and data analysis and presentation. Ethical approval for the study was granted by the ethics committee, Faculty of Medicine Universitas Sebelas Maret in Indonesia. The study was conducted in accordance with the Helsinki Declaration of 1975, as revised in 2008.

## Results

We approached 63 participants via email, short messages, and/or phone calls, and four practitioners from four OR groups did not respond. Therefore, we ultimately analyzed 59 verbatim transcripts of interviews with 36 academics and 23 practitioners.

Our data analysis showed two levels of effect: individual and institutional. Individual effects were categorized into six categories: strengthening of research knowledge, skills, and experience; improvements in understanding of the TB program; increases in motivation for research and self-satisfaction; development/enhancement of individual networking; receipt of recognition; and new opportunities. Institutional effects were categorized into three categories: improvements in research curricula and learning processes, improvements in program management, and development/enhancement of institutional partnerships. Examples of the data analysis processes that consisted of questions, meaning units, codes, categories, and subthemes are shown in Table 1. The coding results are presented in Table 2.

**Table 1.** Derivation of themes: examples of questions, meaning units, codes, categories, and subthemes.

Question	Meaning unit	Code	Category	Subtheme
How would you describe the experience you have gained from your participation in the TORG capacity-building program?	‘OR is a new thing for me. The methods could be applied in the program’ (Participant 44, an academic) ‘I think the positive thing in this program is that the activities [in OR] could be operationalized in the program. This is different from other scientific reviews’ (Participant 52, a practitioner)	Experience of applied research	Strengthening of research knowledge, skills, and experience	Individual effect
	‘I learned how to input data’ (Participant 37, an academic)	Data input and analysis skills		
	‘Yes, this program has great influence. I could see actual [TB-related] problems’ (Participant 15, an academic)	Knowledge of TB control problems	Improvements in understanding of the TB program	
	‘This 10-day workshop enabled me to understand the TB problem in the community …’ (Participant 29, an academic)	Knowledge of the TB situation		
	‘We [the OR team] are frequently in contact; we engage in information sharing and discussion. They [the program-based researchers] consult us about some program-related problems’ (Participant 24, an academic)	Consultation Discussion	Development/enhancement of individual networking	
	‘I included OR teams in education activities at my institution’ (Participant 15, an academic)	Joint activities		
	‘I could give real examples of OR implementation in the research methods course’ (Participant 3, an academic). ‘I included materials for multidrug-resistant TB and TB control in the Social Determinants of Health course’ (Participant 27, an academic)	Embedding materials in teaching	Improvement of research curricula and learning processes	Institutional effect
	‘I could improve some techniques in teaching’ (Participant 47, an academic).	Replicating teaching techniques		
	‘This experience widened my perspective on program implementation. I have understood the TB program problems, but I analyzed the problem during TORG workshops’ (Participant 55, a practitioner)	Analysis of program problems	Improvements in program management	
	‘By conducting monitoring and mapping with the TORG, we solved the problem’ (Participant 8, a practitioner)	Problem solving		
How has the experience influenced your career?	‘Our research received attention from staff at the Ministry of Health’ (Participant 34, a practitioner)	Receipt of attention from the Ministry of Health	Receipt of recognition	Individual effect
	‘The village staff members say that I am a TB expert’ (Participant 9, a practitioner).	Being labeled as a TB expert		
	‘Through TORG, I knew about Ministry of Health research and development. It opened my mind to moving to research and development’ (Participant 43, an academic)	Motivation to pursue a career in research and development	Increases in motivation for research and self-satisfaction	
	‘In 2012, Union gave me an opportunity to conduct OR; the funding was from the KNCV Tuberculosis Foundation in Europe’ (Participant 3, an academic)	Research fellowship	New opportunities	
How has the experience influenced your research interests?	‘The facilitators in the workshop were already professors or held master’s degree … I hold an undergraduate degree. This is one of the reasons for me to continue education’ (Participant 2, a TB program staff)	Motivation for continuing education	Increases in motivation for research and self-satisfaction	
	‘I think OR should be replicated in other districts’ (Participant 22, an academic)	Motivation to conduct OR in other districts		
How have interactions with OR peers influenced communication between your relative institutions?	‘I think that our relationship with the provincial health office is getting better. When they have an event, we are invited to provide input. We are invited to the monitoring evaluation meeting and other meetings’ (Participant 4, an academic)	Academic-program link Joint activities	Development/enhancement of institutional partnerships	Institutional effect

OR = operational research, TB = tuberculosis, TORG = Tuberculosis Operational Research Group.

**Table 2.** Data synthesis: themes, sub-themes, categories, and codes.

Theme	Multiplier effects			
Subtheme	Individual effects			Institutional effects
Category and Codes	Strengthening research knowledge, skills, and experience: Application of research methods theory Knowledge and experience of OR Knowledge of research dissemination plan Scientific writing skills Policy brief writing Data-input and analysis skills Presentation skills Experience of applied research Experience of community research Experience of multidisciplinary research Teamwork Experience of research that improved the program Experience of qualitative research Experience of data analysis and presentation Experience of research dissemination	3. Increases in motivation for research and self-satisfaction: Proud of being a researcher Motivation for continuing education Motivated to conduct OR in other districts Motivation to conduct OR for other programs Motivation to pursue a career in research and development TB as the topic of further research Recharging research New insights in research	5. Receipt of recognition: Appreciation from other researchers Being labeled as a TB expert Receipt of attention from the Ministry of Health Being invited to speak at seminars Being appointed as an internal reviewer Being invited to be a journal reviewer	2.1. Improvements in research curricula and learning processes: Illustration of research for students Embedding materials into the teaching curriculum Enriching student supervision Replication of teaching techniques Case study for in-house training/workshops
				2.2. Improvements in program management: Improvement of TB program/community communication Improvement of quality of TB program data Program innovation Problem analysis Scientific approach to problem solving
	2. Improvements in understanding of the TB program: Additional knowledge of TB problems Knowledge of problems in TB control Knowledge of the real TB situation in the community Widening of the view of TB program implementation	4. Development/enhancement of individual networking: Information sharing Program data sharing Consultation Discussion Networking Additional contacts in universities or programs Personal visits Joint activities	6. New opportunities: Invitation for academic staff to facilitate policy document development Invitation for program staff to be examiners/lecturers Invitation for academic staff to be resource contacts for program seminars Participation in international conferences Receipt of updates about research opportunities Fellowship opportunities Community services Publication in international journals	2.3. Development/enhancement of institutional partnerships: Joint activities between program and university Academic-program link Academic–non governmental organization link Strengthening of existing collaboration Program facilitation to allow students to conduct research in the community Memorandum of understanding between Provincial Health Office and university

OR = operational research, TB = tuberculosis.

### Individual effects

#### Strengthening of research knowledge, skills, and experience

Almost all of the participants (56 of 59) reported improvements in research knowledge, skills, and experience. They described attainment of knowledge and skills, particularly on OR, qualitative research, and data analysis and presentation. Besides this, they reported improvements in research dissemination skills including those involving presentation and writing policy briefs and scientific articles. One of the participants described this as follows:

By participating in the TORG program, I obtained a lot of experience in how to develop a good research proposal and conduct proper data analyses. (Participant 24, an academic)

Another participant stated,

I was introduced to thinking about what has happened and why it happened, then analyze that and write small articles, which I then send to the city government magazine. (Participant 22, a practitioner)

The program also provided experience in conducting applied research and working in multidisciplinary teams.

#### Improvements in understanding of the TB program

The program improved participants’ knowledge, particularly academics’ knowledge, regarding the TB program. This perception was reported by one-third of the academics. One participant stated,

I came to understand that TB is really a community problem. (Participant 57, an academic)

A few practitioners reported attainment of deeper knowledge regarding the TB program. One practitioner stated,

The field study I did automatically add to my understanding of staff in real situations in the TB program. It was an incredible experience. This could help me, as a programmer, to record the real situation in the community. (Participant 59)

#### Increases in motivation for research and self-satisfaction

The experience of participating in the TORG program motivated almost one-third of the participants to conduct further OR. A practitioner from West Java province conducted further OR in a hospital setting. In addition, academics in Central Java were motivated to conduct OR in other districts and for other programs. Remarkably, a practitioner in Papua accepted a research position at the National Institute of Research and Development because the TORG program inspired him about a job as a researcher. In addition, several participants continued their education, with TB as a specific research topic.

Participation in the TORG program improved participants’ motivation for conducting research. The participants were pleased that they had been provided with the opportunity to implement the knowledge regarding research methods that they had obtained from schools. One academic stated,

I feel like my battery has been recharged. (Participant 24)

For practitioners, in particular, the experience of program participation provided new insight regarding research, as expressed in the following participant’s statement:

I feel like I have been in a world other than my own. (Participant 7)

A few participants reported that program participation had induced self-satisfaction because of their contribution to the improvement of the program. One participant stated,

In OR, the study results have the potential to be utilized in the program. As the researcher, I feel proud when that happens. (Participant 24, academic)

#### Development/enhancement of individual networking

Almost half of the participants reported that the TORG program had triggered new individual network connections or intensified existing connections between academic and program staff. Individual networking facilitated academic or TB program-related activities, data collection, and information sharing. One participant described this as follows:

Before I participated in the training, I had no connection to program staff. By participating in the training, I could have discussions, share information, and consult with program staff. (Participant 24, academic)

Another participant stated,

I got to know some university staff. I helped them and their students to conduct research in my district. (Participant 23, a practitioner)

#### Receipt of recognition

Participants described an increase in recognition following their participation in the TORG program and OR. One practitioner (Participant 17) stated that fellow program staff members had recognized her as a TB or research expert, while other practitioners reported appreciation from the Ministry of Health as another form of recognition. From academics’ perspectives, examples of recognition included appreciation from other researchers and invitations to speak at scientific seminars, serve as internal reviewers for research grant selection at universities, and review manuscripts for scientific journals.

#### New opportunities

Networking between academics and practitioners provided additional opportunities. Academics reported receiving invitations to speak at seminars arranged by program staff. In addition, a few participants described receiving fellowship opportunities for TB research from an international donor and invitations to review manuscripts for scientific journals. Moreover, participation in the TB research fellowship provided the opportunity to receive a grant from the international donor for OR concerning tobacco control. In addition, one academic stated that the experience of conducting OR had inspired him to develop a community service project in collaboration with students and the provincial health office.

### Institutional effects

#### Improvements in research curricula and learning processes

This category was revealed from the interviews with academics. Two-thirds of the academics acknowledged improvements in their institutions’ research curricula and learning processes as benefits of OR capacity building. Most academics reported using the field experience in the TORG program as research methodology learning materials for their students. One academic stated,

I frequently use my experience from OR to illustrate good data collection processes. (Participant 22)

Other academics used materials and experiences obtained via the TORG program to develop in-house training for other faculty members at their institutions. In addition, some academics replicated the teaching techniques used in the TORG workshops and field mentoring. Academics acknowledged that their program participation had enriched their supervision of students’ research.

#### Improvements in program management

Both academics and practitioners reported that OR improved program management. Practitioners improved their problem analysis skills via their program participation; therefore, it enhanced their ability to analyze and solve problems in program management. One participant stated,

I knew the problem with the TB control program, but I learned how to analyze it in the TORG. (Participant 55, a practitioner)

In addition, the results of OR broadened program staff members’ perspectives regarding innovation in program management. One participant stated,

Our study results opened up the perspective of the provincial health office regarding health, allowing us to develop program innovations that we only knew about from books. (Participant 58, a practitioner)

#### Development/enhancement of institutional partnerships

The interactions between academic and practitioner established new links between institutions, such as those between academic institutions and provincial health offices or nongovernmental organizations. They also improved existing institutional partnerships between academic institutions and provincial health offices. An academic in Bali province described intensive joint activities between universities and provincial health offices. One academic from West Nusa Tenggara province stated,

Yes, this program strengthened the link [between the provincial health office and university]. Besides strengthening individual links, it also improved our memorandum of understanding with the municipality health office. (Participant 39)

**Figure 1.**
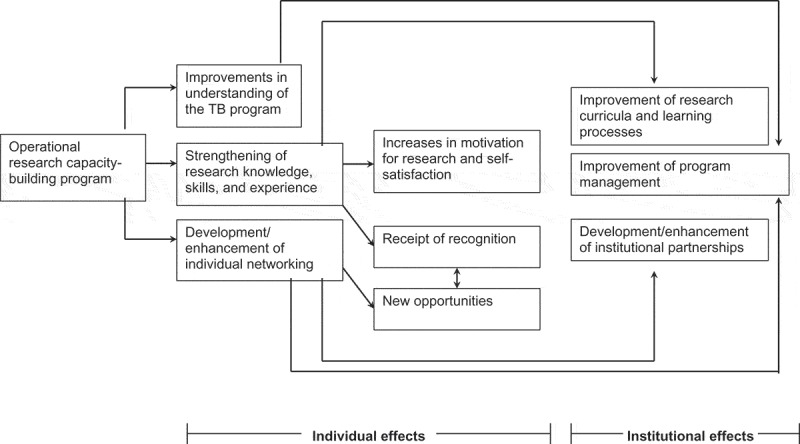
Multiplier effects of the TORG capacity-building program.

The findings also demonstrated a relationship between individual and institutional effects (Figure 1). Perceived improvement in research curricula was related to the enhancement of research knowledge, skills, and experience among academics. In addition, recognition was related to national-level research experience obtained via OR capacity building. Recognition also provided various additional opportunities at local, national, and international levels (e.g. manuscript citations in other international publications or invitations to serve as an internal research grant reviewer, a journal reviewer, or a national-level seminar presenter). These new opportunities, particularly those at a local level, led to improvements in individual networking between academics and practitioners in the same provincial OR group. Perceived program improvement also occurred because of improved understanding regarding problems and solutions, mainly for practitioners. This relationship was facilitated by improvements in communication between academics and practitioners. Improvements in individual networking between the academics and practitioners also intensified institutional networking between local health authorities and universities or health education institutions.

We named the relationship between individual and institutional effects as a ‘social multiplier effect’ as a theme represented the perceived benefit of the TORG capacity-building program from academics’ and practitioners’ perspectives. The theme demonstrated that the program exerted not only individual effects on research knowledge, skills, and experience of the study participants but also other individuals related to institutional effects such as curriculum development and networking.

## Discussions

The study demonstrated that the OR capacity-building program exerted social multiplier effects, indicating that it exerted multiple effects from both academics’ and practitioners’ perspectives. The social multiplier effects theme in our study resulted from our reflection on a connotative meaning of an economic concept of positive social multiplier. The positive social multiplier effect is a spillover of individual behavior to other individuals due to social interactions among them [16]. We meant social multiplier in our findings as the spillover of perceived improved research knowledge and skills of the individuals who directly involved in the TORG program that then was shared among other individuals in their institutions (students and colleagues). The experience also triggers the improvement of the curriculum and/or materials of the research course in the academic institutional, and enhanced the collaboration between the academics and the program.

### Individual effects

The findings showed that the capacity-building program exerted individual effects on the motivation for conducting further OR and improved partnerships between individuals, which is similar to the results of previous evaluations [8,17]. The study also demonstrated that the capacity-building program provided empirical experience of evidence-based TB-control programs and policies. The study informants perceived good examples about research that improved the public health program and influence the policy. Our previous publication explained the impact of this capacity-building program on TB program and policy [9]. In addition, practitioners learned to use evidence to improve TB programs and policy. The finding of perceived improvements in understanding of the TB program is consistent with the results of a study conducted by Naidoo et al. [4]. The effects were reported by both academics and practitioners in the current study, while Naidoo et al. observed the effects only in academics.

### Institutional effects

The results demonstrated three types of institutional effect: improvements in research curricula and learning processes, improvements in program management, and development/enhancement of institutional partnerships. The institutional effects were observed in relation to individual effects. Perceived improvements in research curricula and learning processes reflected improvements in practice among academics. Perceived improvement of data analysis in program management reflected improvements in practice among practitioners. Both academics and practitioners expressed the institutional partnership enhancement effect.

Individual and institutional effects were induced by the application of four experiential learning methods in the TORG capacity building. The participants did not only learn from concepts from lectures, but they also learned from the field works of OR. They received feedback from supervision and mentoring sessions by TORG members. Duration of the TORG capacity-building program (i.e. over 1.5 years) can contribute to the intensity of interactions between the academics and practitioners; hence it can contribute to the institutional networking enhancement.

Boyko et al. [18] identified three levels of effects of knowledge translation: short-term, individual-level effects; medium-term, community- or organizational-level effects; and long-term, system-level effects. The perceived individual- and institutional-level effects observed in the current study reflected short-term effects of knowledge translation such as individual networking and insight regarding TB problem analysis and solutions. The findings also demonstrated medium-term effects of knowledge translation such as that involving academics’ participation in policy document development. Our previous publication showed that when academics’ and practitioners’ approaches to OR are sustained, system-level effects of evidence-based decisions is observed [9].

### Strengths and limitations of the study

The perceived effects of individual networking and institutional partnerships required the context of sufficient intensity of communication between academics and practitioners in OR teams. Moreover, our previous research revealed that communication problems existed within OR teams [9]. One strength of this study was the in-depth nature of the exploration of the benefits of the OR capacity-building program. Moreover, the study examined issues, such as perceived recognition, strengthening of individual partnerships, and enhancement of institutional partnerships, which were not considered in other previous research on OR capacity-building program.

The role of the authors as the Tuberculosis Operational Research Group members on one hand is instrumental to conduct this evaluation. This role facilitated access to information on the sampling frame of informants and helps to familiarize the context and phenomenon. On the other hand, it could introduce biases in data collection and analysis. To minimize this potential bias, we recruited an experienced anthropologist, who was not a TORG member, to conduct interviews and analysis together with AP.

## Conclusion

The TORG capacity-building program exerted multiplier effects at both individual and institutional levels. The academics and practitioners perceived that the program improved individual capacity and motivation in research, and enhanced networking between academics and program staff. In addition, the program triggers the development of institutional links and improved the quality of research curricula in the academic institution and disease control program management of health authorities.

The integration of academic and program staff in the TORG represents a breakthrough in addressing the challenges arising from program staff members’ limited capacity for conducting OR. On the other hand, it allows academics to understand the operational issues of a public health program. Other concerned about inapplicable research due to lacking collaboration between academics and users of research [19]. Therefore, dialogue between academics and practitioners in the OR teams could improve the process involved in knowledge translation.

Therefore, we recommend the intensification and promotion of OR and enhancement of OR capacity building for both academic and program staff as also yield by other [20]. In addition, OR should be included in research methodology curricula for postgraduate public health/disease control programs. Capacity-building models with OR teams that include both academic and program staff should be promoted.

## Paper context

There are current OR capacity-building programs that combine participants from academia and health services. This study found that the OR capacity building program in Indonesia improved individuals’ capacity for conducting OR and the quality of TB program management and public health education. This capacity-building model, in which academics and program staff collaborated within an OR team, should be promoted.
